# STSC-SNN: Spatio-Temporal Synaptic Connection with temporal convolution and attention for spiking neural networks

**DOI:** 10.3389/fnins.2022.1079357

**Published:** 2022-12-23

**Authors:** Chengting Yu, Zheming Gu, Da Li, Gaoang Wang, Aili Wang, Erping Li

**Affiliations:** ^1^College of Information Science and Electronic Engineering, Zhejiang University, Hangzhou, China; ^2^Zhejiang University - University of Illinois at Urbana-Champaign Institute, Zhejiang University, Haining, China

**Keywords:** spiking neural network (SNN), Spatio-Temporal Synaptic Connection (STSC), spike response filter (SRF), Feedforward Lateral Inhibition (FLI), attention mechanism, neuromorphic recognition, backpropagation (BP)

## Abstract

Spiking neural networks (SNNs), as one of the algorithmic models in neuromorphic computing, have gained a great deal of research attention owing to temporal information processing capability, low power consumption, and high biological plausibility. The potential to efficiently extract spatio-temporal features makes it suitable for processing event streams. However, existing synaptic structures in SNNs are almost full-connections or spatial 2D convolution, neither of which can extract temporal dependencies adequately. In this work, we take inspiration from biological synapses and propose a Spatio-Temporal Synaptic Connection SNN (STSC-SNN) model to enhance the spatio-temporal receptive fields of synaptic connections, thereby establishing temporal dependencies across layers. Specifically, we incorporate temporal convolution and attention mechanisms to implement synaptic filtering and gating functions. We show that endowing synaptic models with temporal dependencies can improve the performance of SNNs on classification tasks. In addition, we investigate the impact of performance *via* varied spatial-temporal receptive fields and reevaluate the temporal modules in SNNs. Our approach is tested on neuromorphic datasets, including DVS128 Gesture (gesture recognition), N-MNIST, CIFAR10-DVS (image classification), and SHD (speech digit recognition). The results show that the proposed model outperforms the state-of-the-art accuracy on nearly all datasets.

## 1. Introduction

Spiking neural networks (SNNs) are regarded as the third generation of neural networks (Maass, 1997), with the purpose of addressing the fundamental mysteries of intelligence and the brain by emulating biological neurons and incorporating more biological mechanisms (Roy et al., 2019). The two fundamental components of SNNs are spiking neurons and synapses, which create a hierarchical structure (layers) and subsequently construct a network. SNNs have attracted a significant deal of academic interest in recent years due to their prospective properties, such as the ability to process temporal information (Petro et al., 2019), low power consumption (Roy et al., 2019), and biological interpretability (Gerstner et al., 2014). Currently, SNNs are capable of processing event stream data with low latency and low power (Pei et al., 2019; Gallego et al., 2020). However, there is still a performance gap between SNNs and traditional Artificial Neural Networks (ANNs). Recent SNN training techniques based on surrogate gradients and back-propagation have significantly enhanced the performance of SNNs (Wu et al., 2018; Fang et al., 2021c), while also promoting the further integration of ANNs' modules into SNNs (Hu et al., 2021; Yao et al., 2021; Zheng et al., 2021), greatly accelerating the development of SNNs. However, it remains challenging to connect these computational techniques with the biological properties of SNNs.

Due to the time-dependent correlation of neuron dynamics, it is believed that SNNs naturally process information in both temporal and spatial dimensions (Petro et al., 2019; Roy et al., 2019). Further researches are necessary to harness the spatio-temporal information processing capabilities of SNNs. Combining ANNs' modules has significantly increased the performance of SNNs in several research studies. In terms of spatial information processing, CSNN (Xu et al., 2018) was the first to validate the application of convolution structure on SNNs, followed by the proposal of NeuNorm to improve SNNs' usage of convolution through auxiliary neurons (Wu et al., 2019). In the time dimension, Zheng et al. (2021) implements the time-dependent batch normalization (tdBN) module to tackle the issue of gradient vanishing and threshold balancing, and Yao et al. (2021) uses the Squeeze-and-Excitation (SE) block (Hu et al., 2018) to realize the attention distribution of the temporal dimension in order to improve the temporal feature extraction. Notably, Zhu et al. (2022) proposes Temporal-Channel Joint Attention (TCJA) to concurrently process input in both temporal and spatial dimensions, which is a significant effort for SNNs' spatio-temporal feature extraction. These studies effectively improve the performance of SNNs by transplanting established ANNs' modules and methodologies. However, applying these computational modules to SNNs from the standpoint of deep learning dilutes the fundamental biological interpretability, bringing SNNs closer to a mix of existing concepts in machine learning, such as recurrent neural networks (RNNs), binary neural networks (BNNs), and quantization networks.

From a biological standpoint, some works focus on the synapse models, investigating the potential of SNNs in respect of connection modes and information transmission. Shrestha and Orchard (2018), Fang et al. (2020a), and Yu et al. (2022) integrate impulse response models with synaptic dynamics, hence enhancing the temporal information representation of SNNs; Cheng et al. (2020) implements intra-layer lateral inhibitory connections to improve the noise tolerance of SNNs; from the standpoint of synaptic plasticity, Zhang and Li (2019) and Bellec et al. (2020) introduce bio-plausible training algorithms as an alternative to back-propagation (BP), allowing for lower-power training. Experiments revealed that the synaptic models of SNNs have a great deal of space for modification and refinement in order to handle spatio-temporal data better (Fang et al., 2020a). We propose a Spatio-Temporal Synaptic Connection (STSC) module for this reason.

**Figure 1:**
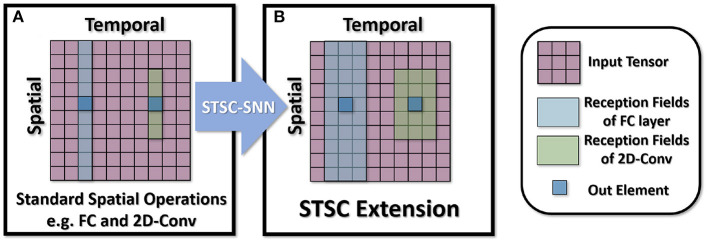
Illustration of receptive fields in synaptic connections. **(A)** The receptive fields of typical spatial operations used in SNNs, e.g., fully-connected layers (full) and 2D convolutional layers (sparse); **(B)** The STSC modules proposed to extend spatial operations with spatio-temporal receptive fields.

Based on the notion of spatio-temporal receptive fields, the structural features of dendritic branches (Letellier et al., 2019) and feedforward lateral inhibition (Luo, 2021) motivate this study. By merging the ANNs' computation modules (temporal convolutions and attention mechanisms) with SNNs, we propose the STSC module, consisting of Temporal Response Filter (TRF) module and Feedforward Lateral Inhibition (FLI) module. As shown in Figure 1, the STSC can be attached to spatial operations to expand the spatio-temporal receptive fields of synaptic connections, hence facilitating the extraction of spatio-temporal features.

The main contributions of this work are summarized as follows:

- We propose STSC-SNN to implement synaptic connections with extra temporal dependencies and enhance the SNNs' capacity to handle temporal information. To the best of our knowledge, this study is the first to propose the idea of synaptic connections with spatio-temporal receptive fields in SNNs and to investigate the influence of synaptic temporal dependencies in SNNs.
- Inspired by biological synapses, we propose two plug-and-play blocks: Temporal Response Filter (TRF) and Feedforward Lateral Inhibition (FLI), which perform temporal convolution and attention operations and can be simply implemented into deep learning frameworks for performance improvements.
- On neuromorphic datasets, DVS128 Gesture, SHD, N-MNIST, and CIFAR10-DVS, we have produced positive results. Specifically, we acquire 92.36% accuracy on SHD with a simple fully-connected structure, which is a great improvement above the 91.08% results obtained with recurrent structure and reaches performance comparable to ANNs.

## 2. Methods and materials

### 2.1. The networks components and training methods in spiking neural networks

#### 2.1.1. Learning algorithms for SNNs

In recent years, many works have explored the learning algorithms of SNNs, which can be generally categorized as biologically inspired approaches (Diehl and Cook, 2015; Zhang and Li, 2019; Bellec et al., 2020), ANN-to-SNN conversion methods (Orchard et al., 2015; Sengupta et al., 2019; Han et al., 2020), and surrogate-based direct training methods (Wu et al., 2018; Neftci et al., 2019; Fang et al., 2021c). Bio-inspired algorithms are represented by spike-timing-dependent plasticity (STDP; Diehl and Cook, 2015; Vaila et al., 2020) which performs local learning through the learning rules of synaptic plasticity, and is often used in unsupervised learning. Some biologically inspired algorithms that integrate local and global learning also provide more efficient training methods, such as ST-RSBP (Zhang and Li, 2019) and e-prop (Bellec et al., 2020). These methods further enhance the low power consumption and biological interpretability of SNNs, although they are not yet capable of producing competitive outcomes in refined tasks like pattern recognition. The ANN-SNN conversion strategies aim to convert the trained ANNs into SNNs and then exploit the low power consumption of SNNs to improve inference (Orchard et al., 2015; Sengupta et al., 2019; Han et al., 2020; Zhang et al., 2021b). The main advantage of conversion methods is that they can directly achieve larger-scale and higher-performance tasks inherited from ANNs. However, they disregard the temporal dynamics and temporal interdependence of spiking neurons and always need hundreds of time steps to approximate the performance of trained ANNs; consequently, hybrid training methods that further optimize converted SNNs were developed (Rathi et al., 2020; Rathi and Roy, 2021; Zhang et al., 2021a). Direct training methods utilize surrogate gradients to tackle the issue of non-differentiable spike activity (Wu et al., 2018), allowing error back-propagation (BP) through time to interface the gradient descent directly on SNNs for training. Those BP-based methods show strong potential to achieve high accuracy in a few timesteps by making full use of spatio-temporal information (Wu et al., 2019; Fang et al., 2021c). However, more research is required to determine how to better extract spatio-temporal features for enhanced processing of spatio-temporal data; this is what we want to contribute.

#### 2.1.2. Attention modules in SNNs

The attention mechanism distributes attention preferentially to the most informative input components, which could be interpreted as the sensitivity of various inputs. The SE block (Hu et al., 2018) offers an efficient attention approach to improve representations in ANNs. Xie et al. (2016) and Kundu et al. (2021) introduced spatial-wise attention in SNNs; then, TA-SNN (Yao et al., 2021) developed a temporal-wise attention mechanism in SNNs by assigning attention factors to each input frame; more subsequently, TCJA (Zhu et al., 2022) added a channel-wise attention module and proposed temporal-channel joint attention. These studies demonstrate the usefulness of attention mechanisms in SNNs by achieving state-of-the-art results on various datasets. Moreover, based on these investigations, it is desirable to study other correlations between the attention mechanism and the biological nature of SNNs, which is the objective of our research. We employ the attention module as a feedforward lateral inhibitory connection (Luo, 2021), which develops a gating mechanism for the synapse model, and enables non-linear computation by the synapse.

#### 2.1.3. Synaptic models in SNNs

As one of the fundamental components of SNN, the synaptic model has drawn the interest of several researchers. Shrestha and Orchard (2018), Fang et al. (2020a), and Yu et al. (2022) established temporal relationships between response post-synaptic currents and input pre-synaptic spikes, therefore improving temporal expressiveness. Those temporal relationships are the extension of fully-connected synapses which are based on the assumption that there is only one connection between two neurons. Nevertheless, synaptic connections are often complex, and there are typically many paths connecting the axons and dendrites of neurons (Letellier et al., 2019; Luo, 2021). We apply temporal convolution to describe the more sophisticated impulse response model and generate time-dependent post-synaptic currents, taking into consideration biological features and computational simplicity.

### 2.2. Frame-based representation

Event steam consists of both a spatial and a temporal dimension, with the spatial dimension expandable to higher dimensions depending on the data type. The spatial dimension of event streams based on sound data is typically one-dimensional, corresponding to different frequency channels; whereas the spatial dimension of event streams based on image data is typically three-dimensional, consisting of coordinates representing spatial positions and polarities representing brightness changes. The binary spike pattern is represented by the tensor *E*∈*B*^*T*^′ × *S*, where *T*′ represents the original resolution in the temporal dimension, and *S* represents the resolution in the spatial dimension. For a frame with a time span of Δ*t*, the events in the time interval *t*′∈[(*t*−1) × Δ*t, t*×Δ*t*) can be mapped to the network input **X**^0^ at time *t* by


(1) $$\begin{array}{l} x^{0}\left(t\right)=q\left(\left\{E\left(t^{\prime }\right)|t^{\prime }\in \left[\left(t-1\right)\times \Delta t,t\times \Delta t\right)\right\}\right) \end{array}$$

where *t*∈{1, 2, ..., *T*} is timesteps, and the aggregation function *q*(·) could be chosen as non-polarity aggregation (Massa et al., 2020), accumulate aggregation (Deng et al., 2020), AND aggregation (He et al., 2020), etc. Here, we choose to accumulate all event streams inside a frame.

### 2.3. Spiking neurons in SNNs

The Leaky-Integrate-and-Fire (LIF) model was introduced as an extremely simplified model of biological neurons (Dayan and Abbott, 2005), which has the essential qualities of potential integrating, leaking, and spike firing. The LIF model is used extensively in SNNs and neuromorphic engineering because of its ability to recreate essential neural functions at a minimal cost of computation. The LIF model is defined in the differential form, as


(2) $$\begin{array}{l} \tau \frac{dv\left(t\right)}{dt}=-v\left(t\right)+I\left(t\right) \end{array}$$

where *v*(*t*) is the membrane potential of the neuron at time *t*, *I*(*t*) is the integrated current input from the pre-synaptic neuron at time *t*, and τ is the time constant that governs the pace of potential change. Solving the differential equation directly will incur additional costs. STBP (Wu et al., 2018) employs a simplified iterative representation and implements the LIF model on the Pytorch framework (Paszke et al., 2019), which supports the integration of SNNs and standard ANNs' modules and significantly speeds the construction of BP-based SNNs and training techniques. The explicit iterative LIF is expressed as


(3) $$\begin{array}{l} {\text{V}}^{l}\left(t\right)=\left(1-\frac{1}{\tau }\right)\times {\text{V}}^{l}\left(t-1\right)\times \left(1-{\text{S}}^{l}\left(t-1\right)\right)+{\text{I}}^{l}\left(t\right) \end{array}$$

(4) $$\begin{array}{l} {\text{S}}^{l}\left(t\right)=\Theta \left({\text{V}}^{l}\left(t\right)-V_{th}\right) \end{array}$$

where *l* and *t* are indices of layer and time, τ is the time constant, **V** is the membrane potential, *V*_*th*_ is the threshold constant, **S** is the binary tensor of spikes, **I** is the input from the preceding layer, and Θ(·) is the Heaviside step function that satisfies Θ(*x*) = 1 when *x*≥0, otherwise Θ(*x*) = 0. Noting that the firing process, Θ(·), is not differentiable, surrogate methods are often utilized in SNNs' direct training to achieve error propagation by creating various pseudo-derivatives for Θ(·) (Neftci et al., 2019). Experiments conducted by STBP (Wu et al., 2018) demonstrate that the performance of each surrogate gradient is comparable. This work leverages arc tangent (ATan) as the pseudo-derivative of Θ(·), which is well-supported in the SpikingJelly framework (Fang et al., 2020b).

### 2.4. Spatio-temporal receptive fields in SNNs

The receptive field is often used to comprehend convolution procedures. In the process of convolution, the receptive fields describe the range of the nearby input for identifying an output element, i.e., how much spatial neighboring position it can perceive. For static pictures, the receptive field could explain the projection range of the convolution operations and aid in the comprehension of the spatial feature extraction procedure. Similarly, the concept of receptive fields could be applied to event streams (or dynamic images) with an additional temporal dimension. This work leverages the concept of spatio-temporal receptive fields to aid comprehension of SNNs' spatio-temporal feature extraction procedure. As shown in Figure 1A, typical synaptic connections employ 2D convolution, pooling, full-connections, and other inter-layer computations to process information in the spatial dimension, which we refer to as spatial operations, and their receptive fields are restricted to the spatial dimension. To strengthen the spatio-temporal information processing capabilities of SNNs, it is essential to expand the receptive fields of these spatial operations into the temporal dimension.

### 2.5. Spatio-Temporal Synaptic Connection for SNNs

**Figure 2:**
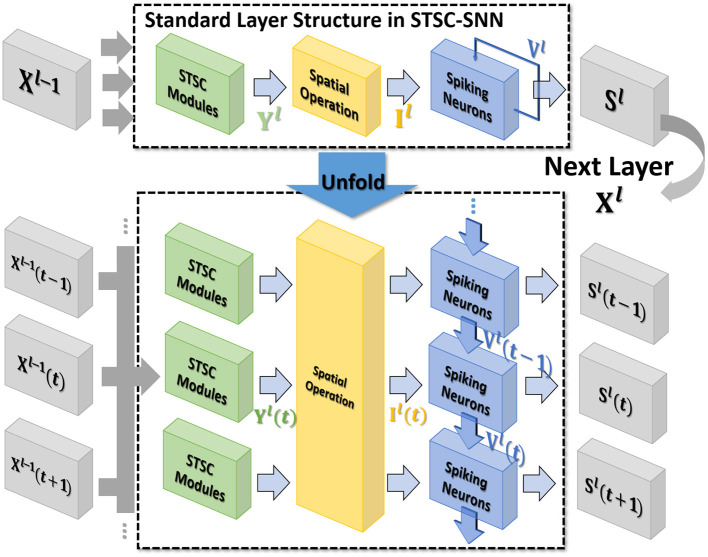
The standard layer inserted with the STSC module and its unfolded formulation. Note that all parameters are shared at all timesteps. STSC modules are set before spatial operations to process the latest temporal information.

**Figure 3:**
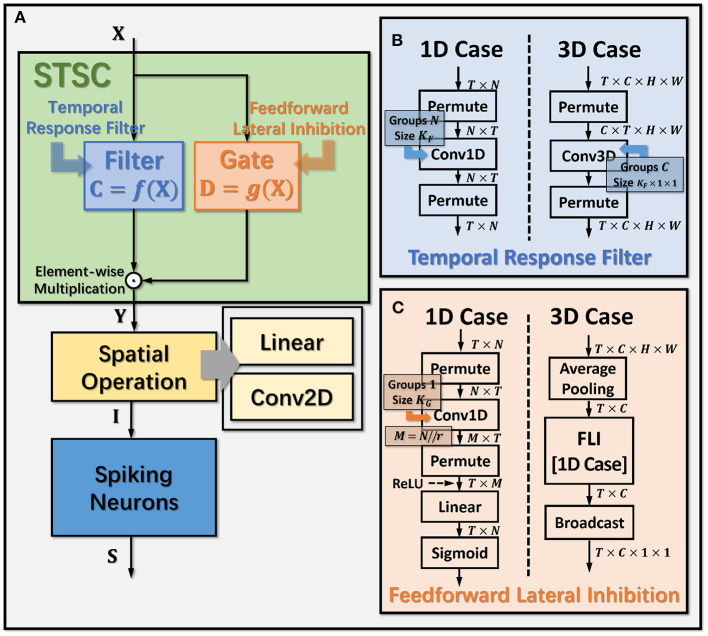
Operation details of STSC modules. **(A)** Connection Implementation between TRF, FLI, and Spatial Operations, where ⊙ denotes broadcast element-wise multiplication; **(B, C)** Tensor Computations in TRF and FLI modules, where *T, C, H, W* refer to tensor dimensions of time, channel, height, and width.

**Figure 4:**
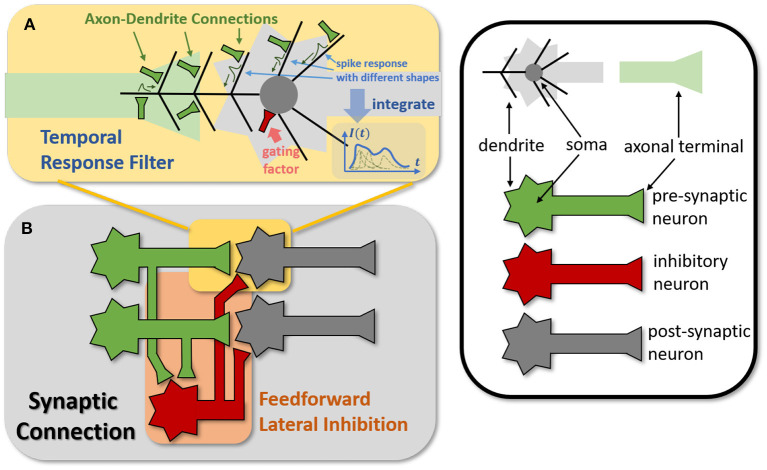
Modeling TRF and FLI modules from biological circuits into computational models. **(A)** The motivation of TRF that integrating multi-compartment axon-dendrite connections with different spike responses into a single linear response filter. **(B)** The function of FLI that transmitting the spike activities of pre-synaptic neurons into inhibitory neurons to obtain gating factors.

**Figure 5:**
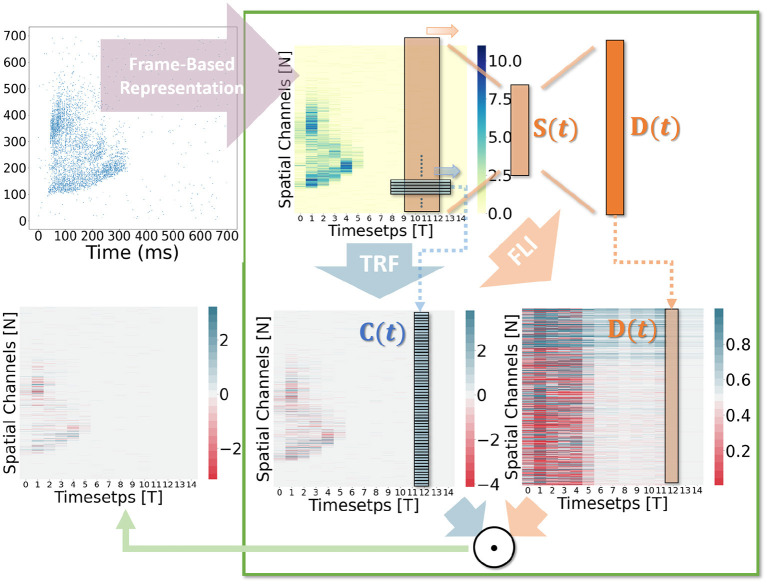
Computation visualization. The input is an audio sample from the SHD dataset.

In general, the processing of temporal information in SNNs is attributed to spiking neurons since their dynamic model has a natural dependence on the temporal dimension; however, the level of this dependence is primarily reliant on the degree of neural complexity, while the LIF neurons only support very weak temporal linkages. Not just in neurons, but also in biological synapses, a great deal of the processing of latent temporal characteristics occurs (Letellier et al., 2019; Luo, 2021). This work focus on using temporal dimension operations in SNNs to broaden the spatio-temporal receptive fields of synapses, to enhance the spatio-temporal feature extractions of SNNs. Temporal operations are calculations connected to the time dimension, and they are contained in a pluggable module, referred to as the Spatio-Temporal Synaptic Connection (STSC). The STSC module is designed to be placed before spatial operations in order to aggregate temporal information and enlarge the spatio-temporal receptive fields while maintaining the original spatial operations (see Figure 2). The STSC module consists of two modules: Temporal Response Filter and Feedforward Lateral Inhibition, which carry the filtering and gating mechanisms of the synaptic model, respectively (see Figure 3). The two modules receive **X** as input tensor and conduct the operations **C** = *f*(**X**) and **D** = *g*(**X**), followed by element-wise product to produce output **Y** = **C**⊙**D** (see Figure 3A). As shown in Figure 4, our starting point is to reconstruct the synaptic connection of the neural circuit in order to get a simplified computing model comprised of TRF and FLI modules while preserving the temporal operations of synaptic computing to the greatest extent. Consequently, our synaptic model provides a further appropriate imitation of biological neural networks with biological interpretability; it also enhances the model's capabilities for spatio-temporal feature extraction by incorporating additional temporal computations. Figure 5 provides an illustration of the model's dataflow.

#### 2.5.1. Temporal response filter

The synapses in biological neural networks are intricate. The complexity of synapses is not only reflected in the non-topological spatial relationship of synapses (how neurons connect with one another) but also in the complicated temporal dependency of spike transmission (how neurons communicate with one another; Letellier et al., 2019). The axon terminals and dendrites of a pair of connected neurons are typically connected by multiple pathways, as shown in Figure 4A. Since the spike responses (including time delays and kernel shapes) in the various pathways are diverse from one another, this implies that more complicated temporal dynamic dependencies can be shaped among them (Luo, 2021). This work assumes that the diverse spike responses of the various pathways can be combined into a single, adaptable response (Figure 4A). Thereby, we propose the Temporal Response Filter (TRF) to establish the integrated linear response of spikes over time by employing convolution in the time dimension, in order to expand the temporal receptive field in the most direct way. TRF offers a filtering path for STSC with temporal convolutions (see Figure 3). Figure 3B depicts the specific implementation of TRF. In detail, as for the 2D spatiotemporal tensor in the fully-connected structure, it performs temporal depth-wise 1D convolution independently on each spatial channel and generates an output tensor of the same size. To ensure that all spatial elements inside a channel have the same temporal response, for the 4D spatiotemporal tensor in the convolutional structure, temporal depth-wise 3D convolution is performed on each channel with kernel size of *K*_*G*_×1 × 1. Mathematically, the filter operation is denoted as *f*(·), and it performs **C** = *f*(**X**) with input **X** and output **C** having the same size as *R*^*T*×*N*^ or *R*^*T*×*C*×*H*×*W*^. Depending on the spatial dimension (1D or 3D) of the input **X**, the following calculation formulae apply:As for the 1D case,


(5) $$\begin{array}{l} \text{C}\left(t,n\right)=\overset{\frac{K_{F}-1}{2}}{\underset{t_{f}=-\frac{K_{F}-1}{2}}{\sum }}{\text{W}}_{t_{f},n}^{F}\times \text{X}\left(t-t_{f},n\right) \end{array}$$

As for the 3D case,


(6) $$\begin{array}{l} \text{C}\left(t,c,h,w\right)=\overset{\frac{K_{F}-1}{2}}{\underset{t_{f}=-\frac{K_{F}-1}{2}}{\sum }}{\text{W}}_{t_{f},c}^{F}\times \text{X}\left(t-t_{f},c,h,w\right) \end{array}$$

where *n*,*c*,*h*, and *w* are spatial location indices and *t* is a time index. *K*_*F*_ denotes the kernel size of the temporal convolution, which is equal to the temporal receptive fields of TRF. The padding of the convolution is set to $\frac{K_{F}-1}{2}$ for maintaining the same size.

#### 2.5.2. Feedforward lateral inhibition

The mechanisms of feedforward lateral inhibition mechanisms exist in biological neural networks (Luo, 2021), which construct a lateral route to suppress feedforward input. To further boost synaptic expression, we attempted to emulate the feedforward lateral inhibition mechanism (see Figure 4B). We devised a computational strategy that replicates the function of inhibitory neurons by exploiting the near-time spike patterns of pre-synaptic neurons to determine the inhibition coefficients, also known as gating factors, which are utilized to regulate the transmission intensity of signals. While the TRF module based on temporal convolution is a straightforward linear computation, the FLI module incorporates non-linear components, resulting in a non-linear relationship between temporal dependencies that improves spatio-temporal feature extraction. We notice that the function of this structure closely resembles that of the attention module; therefore, we refer to the attention blocks (Hu et al., 2018; Yao et al., 2021; Zhu et al., 2022), and propose the FLI module to replicate the gating mechanism in synaptic connections. The module details are shown in Figure 3C. Regarding the 2D spatiotemporal tensor in the fully-connected structure, temporal-wise 1D convolution is utilized first to extract temporal features, followed by linear combination through sigmoid to acquire the gating coefficients (see Figure 3C). As for the 4D spatiotemporal tensor in the convolutional structure, spatial-wise average pooling is first conducted to obtain the channel-wise spatial sparsity of spikes; then, the 1D case FLI is performed. Finally, channel-wise gating factors are computed and transmitted to each channel's spatial locations. Mathematically, gating is denoted as *g*(·), and **X** is the input tensor of size *R*^*T*×*N*^ or *R*^*T*×*C*×*H*×*W*^, **D** = *f*(**X**) is the output gating factors with values in the range (0, 1) that have the same shape with **X**. Depending on the spatial dimension (1D or 3D) of the input **X**, the following calculation equations apply:As for the 1D case:


(7) $$\begin{array}{l} \text{S}\left(t,m\right)=\overset{\frac{K_{G}-1}{2}}{\underset{t_{g}=-\frac{K_{G}-1}{2}}{\sum }}\overset{N}{\underset{n=1}{\sum }}{\text{W}}_{t_{g},n,m}^{G1}\times \text{X}\left(t-t_{g},n\right) \end{array}$$

(8) $$\begin{array}{l} \text{D}\left(t,n\right)=\text{S}igmoi\text{d}\left(\overset{M}{\underset{m=1}{\sum }}{\text{W}}_{m,n}^{G2}\times \text{R}eL\text{U}\left(\text{S}\left(t,m\right)\right)\right) \end{array}$$

As for the 3D case:


(9) $$\begin{array}{l} \hat{\text{X}}\left(t,c\right)=\frac{1}{H\times W}\overset{H}{\underset{h=1}{\sum }}\overset{W}{\underset{w=1}{\sum }}\text{X}\left(t,c,h,w\right) \end{array}$$

(10) $$\hat{D}=g_{1D}\left(\hat{X}\right)$$

(11) $$D\left(t,c,h,w\right)=\;\hat{D}\left(t,c\right)\;\;\;\;for\;\forall h\in H,\forall w\in W$$

where *n*,*c*,*h* and *w* are spatial location indices, *t* is a time index, *m* is the index of the intermediate feature tensor *S* with spatial dimension *M*. *M* is determined by the spatial sizes *N* with reduction ratio *r*, as $\frac{N}{r}$. *K*_*G*_ denotes the kernel size of the convolution, which is equivalent to the receptive fields of FLI. The padding of the convolution is set to $\frac{K_{G}-1}{2}$ for maintaining the same size. Notably, in order to reduce computational cost in the 3D case, we employ the same gating factor for all *H*×*W* neurons in each channel, feed the spiking space sparsity into the FLI module (Equation 9), and broadcast the output to all neurons inside the channel (Equation 11).

### 2.6. Training framework

Denote the simulating timesteps as *T*, size of output layers as *L*_*out*_ and classes number as *C*, we utilize the voting strategy (Wu et al., 2019) to decode the network output $O\in B^{T\times L_{out}}$ with the constant voting matrix $M\in R^{C\times L_{out}}$. The loss function is defined by the mean squared error (MSE), as


(12) $$L={\left\|y_{i}-\frac{1}{T}\sum M_{i,n}O\left(t,n\right)\right\|}^{2}$$

where **y** is the one-hot target, with **y**_*l*_ = 1 for target class *l*, and **y**_*i*_ = 0 for *i*≠*l*. The predicted label *l*_*p*_ is then given by $l_{p}=argmax_{i}\frac{1}{T}\sum M_{i,n}O\left(t,n\right)$ for evaluation. In the experiment, we adopted the simplest voting strategy and obtained ∑*M*_*i, n*_*O*(*t, n*) through average pooling.

## 3. Experiments

### 3.1. Experiment setup

#### 3.1.1. Datasets

We evaluate the classification performance of STSC-SNN on a variety of neuromorphic datasets, including DVS128 Gesture (Amir et al., 2017; gesture recognition), N-MNIST (Orchard et al., 2015), CIFAR10-DVS (Li et al., 2017; image classification), and SHD (Cramer et al., 2020; speech digit recognition), all of which are event datasets but are generated using different methods. DVS128 Gesture is a gesture recognition dataset that uses DVS cameras to record actual human gestures. The event-based image datasets, N-MNIST and CIFAR10-DVS, are converted from the static dataset by using DVS cameras to scan each sample. Spiking Heidelberg Digits (SHD) is a spike-based speech dataset consisting of English and German spoken digits transformed from the audio recordings using an artificial inner ear model.

#### 3.1.2. Settings

Table 1 summarizes the experimental details of the SNNs training process. We use the SpikingJelly (Fang et al., 2020b) and Pytorch (Paszke et al., 2019) frameworks to develop and evaluate SNNs. We utilize the Adam optimizer (Kingma and Ba, 2014) to accelerate the training process. Table 2 displays the respective hyper-parameters and Table 3 displays the network architectures for different datasets. The adequate numbers of epochs are utilized to assure the models' steady convergence (Table 2). The settings of network architecture are various in related works, and the network structures we use (Table 3) have been proven to perform quite well on each dataset [specifically, SHD refers to TA-SNN (Yao et al., 2021), N-MNIST refers to PLIF (Fang et al., 2021c), CIFAR10-DVS and DVS128 Gesture refer to TCJA-SNN (Zhu et al., 2022)]. All Conv2d layers are set as kernel size = 3, stride = 1, and padding = 1, followed by batch normalization (BN) layers. The voting layers are implemented using average pooling for classification robustness (Fang et al., 2021c).

**Table 1:** Experimental details.

**Datasets**	**SHD and CIFAR10-DVS and DVS128 Gesture and N-MNIST**
Representation	Frames with accumulative aggregation
Learning algorithm	STBP (Wu et al., 2018) and BPTT
Surrogate gradient	ATan (Fang et al., 2021c)
Loss function	Voting (Wu et al., 2019) and MSE
Frameworks	SpikingJelly and Pytorch

**Table 2:** Hyper-parameter setting.

**Hyper parameter**	**SHD**	**N-MNIST**	**CIFAR10-DVS**	**DVS128 Gesture**
Epoch	200	300	1,000	1,000
Batch size	256	16	16	16
Learning rate	1e-4	1e-3	1e-3	1e-3
*T*	15	10	10	20
τ	10	2	2	2
*V*_*th*_	0.3	1.0	1.0	1.0
*K*_*F*_	5	3	3	3
*K*_*G*_	3	3	3	5
*r*	1	1	2	2

**Table 3:** Network structure.

**Dataset**	**Network structure**
DVS128 Gesture	Input-128C3-MP2-128C3-MP2-128C3- MP2-128C3-MP2-128C3-MP2-0.5DP-512FC-0.5DP-110FC-Voting-11
CIFAR10-DVS	Input-64C3-128C3-AP2-256C3-256C3- AP2-512C3-512C3-AP2-512C3-512C3- AP2-100FC-Voting-10
SHD	Input-128FC-128FC-100FC-Voting-20
N-MNIST	Input-128C3-AP2-128C3-AP2-0.5DP- 2048FC-0.5DP-100FC-Voting-10

*x*C*y*/MP*y*/AP*y* denotes the Conv2D/MaxPooling/AvgPooling layer with output channel = *x* and kernel size = *y*×*y*, *n*F*C* denotes the fully connected layer with output feature = *n*, *m*DP is the spiking dropout layer with dropout ratio *m*. The spiking neurons are added behind all *x*C*y* and *n*FC. The STSC modules are inserted before all *x*C*y* on DVS128 Gesture, CIFAR10-DVS, and N-MNIST; while inserted before all *n*FC on SHD.

### 3.2. Comparison with existing SOTA works

Table 4 shows the performance comparison of the proposed methods (STSC-SNN with TRF and FLI) and other competing methods on neuromorphic datasets, N-MNIST, CIFAR10-DVS, DVS128 Gesture, and SHD. The trials indicate that STSC-SNN can achieve similar or even better results under the same conditions as SOTAs. These results demonstrate that our work is comparable to SOTAs' and reveal that our models inspired by biological synapses are practical and feasible. As shown in Table 4, we achieve the highest accuracy on all datasets except CIFAR10-DVS. The SOTA results implemented in CIFAR10-DVS are based on the work of TET (Deng et al., 2022), which proposes a new loss function to enable the model to converge on a flatter local minimum with generalizability; TCJA (Zhu et al., 2022) also demonstrates its efficacy on CIFAR10-DVS. To preserve the consistency of this work, we continue to utilize MSE (Equation 12) as the loss function, and outperform the comparable result. Notably, the experiments on SHD show that we have enhanced the vanilla SNN from 78.71 to 92.36% using STSC (with 65 epochs), which is the state-of-the-art result compared to the highest available result (91.08% by TA-SNN). Moreover, it is a significant improvement that even reaches the best result achieved by ANNs on this dataset [92.4% by CNN (Cramer et al., 2020)].

**Table 4:** Performance comparison between the proposed method and the state-of-the-art methods on different datasets.

**Method**	**SHD**		**N-MNIST**		**CIFAR10-DVS**		**DVS128 Gesture**	
	**T**	**Acc. (%)**	**T**	**Acc. (%)**	**T**	**Acc. (%)**	**T**	**Acc. (%)**
Slayer (Shrestha and Orchard, 2018)	-	-	300	99.22	-	-	1,600	93.4
HATS (Sironi et al., 2018)	-	-	-	99.10	-	52.4	-	-
DART (Ramesh et al., 2019)	-	-	-	97.95	-	65.8	-	-
NeuNorm (Wu et al., 2019)	-	-	-	99.53	-	60.5	-	-
Rollout (Kugele et al., 2020)	-	-	32	99.57	48	66.97	240	97.27 (10 classes)
DECOLLE (Kaiser et al., 2020)	-	-	-	-	-	-	500	95.7
LISNN (Cheng et al., 2020)	-	-	20	99.45	-	-	-	-
tdBN (Zheng et al., 2021)	-	-	-	-	10	67.8	40	96.87
LIAF-Net (Wu et al., 2021)		-	-	-	10	70.40	60	97.56
PLIF (Fang et al., 2021c)	-	-	10	99.61	20	74.80	20	97.57
LIF RSNN (Cramer et al., 2020)	2,000	73.3	-	-	-	-	-	-
Hetero. RSNN (Perez-Nieves et al., 2021)	-	83.5	-	97.5	-	-	-	82.9
RELU SRNN (Yin et al., 2020)	250	88.93	-	-	-	-	-	-
Adaptive SRNN (Yin et al., 2021)	250	90.4	-	-	-	-	-	-
SEW-ResNet (Fang et al., 2021b)	-	-	-	-	16	74.4	16	97.92
TA-SNN (Yao et al., 2021)	15	91.08	-	-	10	72.00	20	98.61
Dspike (Li et al., 2021)	-	-	-	-	10	75.45	-	-
SALT (Kim and Panda, 2021)		-	-	-	20	67.1	-	-
TET (Deng et al., 2022)	-	-	-	-	10	**83.32**	-	-
DSR (Meng et al., 2022)	-	-	-	-	10	77.41	-	-
TCJA (Zhu et al., 2022)	-	-	-	-	10	80.7(MSE) **83.3(TET)**	20	**99.0**
STSC (this work)	15	**92.36**	10	**99.64**	10	**81.4(MSE)**	20	**98.96**

The bold values indicate the best outcomes for each dataset.

The SHD dataset contains rich temporal information, which challenges the model's capacity to extract temporal features (Cramer et al., 2020); hence, there is considerable effort required to develop SNN models using recurrent structures (Cramer et al., 2020; Yin et al., 2020, 2021; Perez-Nieves et al., 2021). Based on the recurrent structure, TA-SNN employs temporal-wise attention and a particular LIF neuron [LIAF by Wu et al. (2021) that directly transmits membrane potential] to get an excellent result on SHD (91.08%), outperforming LSTM [89% by Cramer et al. (2020)] but falling short of the result (92.4%) produced by CNN processing (directly as 2D image input). In contrast, instead of the recurrent layers, we use a simple fully-connected network with two hidden layers and successfully obtain the SOTA result by adding the proposed STSC module. For the first time, our model obtained CNN-like performance on the SHD dataset, which represents a substantial effort to illustrate the SNNs' potential.

### 3.3. Control experiments and ablation study

**Figure 6:**
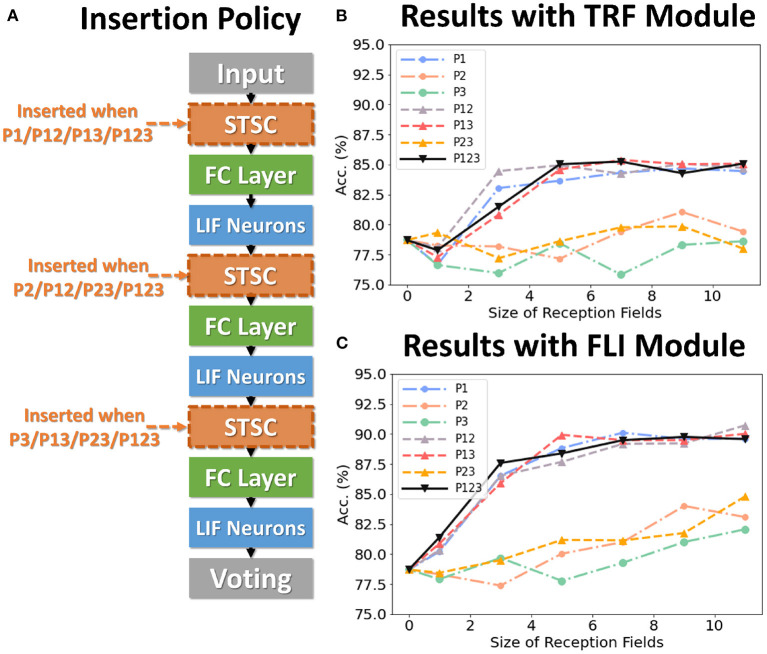
Control experiments on SHD. **(A)** Policies of inserting STSC layers; **(B)** Accuracy comparison of TRF module *via* different RFs; **(C)** Accuracy comparison of FLI module *via* different RFs.

**Figure 7:**
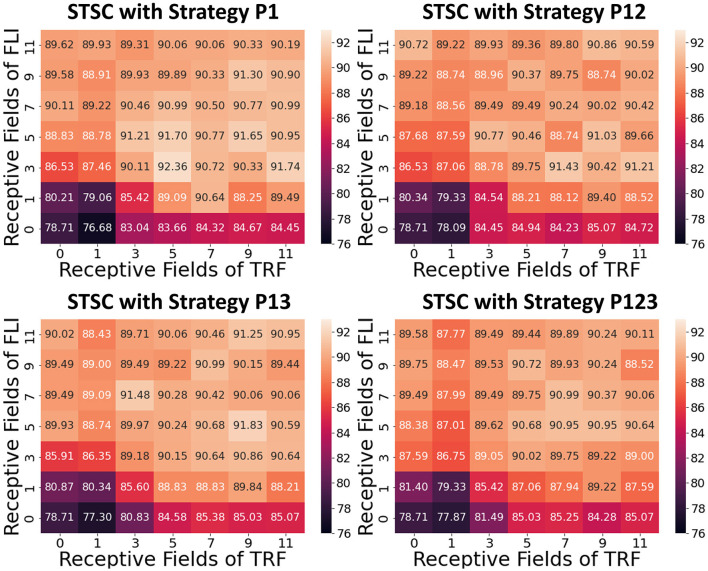
Influence of receptive fields on SHD. The different experimental results *via* RFs with P1/P12/P13/P123.

To analyze the impact of each component on performance, we conduct control experiments on SHD. The SHD experiment is based on the fully-connected (FC) structure (see Table 3), with STSC modules strategically placed in front of the FC layers. There are seven insertion strategies designated P1, P2, P3, P12, P13, P23, and P123 (see Figure 6A). Figures 6B, C show the effect of varying receiving fields when TRF and FLI are used individually. TRF reaches 85.38% at P13 and RF = 7, while FLI reaches 90.72% at P12 and RF = 11. Comparing the two modules reveals that the FLI module plays a major role in performance improvement; thus, it is crucial to offer a gating mechanism that introduces non-linear expressions to FC layers. Figures 6B, C demonstrate that when the FLI or TRF modules are positioned in the first layer (P1/P12/P13/P123), they have a greater impact on performance than when they are positioned in the deep layer (P2/P3/P23). This suggests that the extraction of temporal features is more advantageous in shallow layers. As shown in Figure 7, we evaluated the impact of varying STSC receptive fields on SHD performance. Notably, raising the receptive field suitably will increase performance, whereas an overly broad receptive field setting would reduce accuracy. We claim the performance drop is a result of the model's excessive expressive capacity, which overfits the train data. This phenomenon is analogous to the usage of spatial 2D convolutions, in which the kernel size must be carefully determined. Figure 7 indicates that the combination of TRF and FLI modules improves performance, demonstrating their complementarity. Under the P1 strategy, setting TRF's RF = 5 and FLI's RF = 3 yields the best result of 92.36%, with just one STSC added after the input layer.

### 3.4. Analysis of temporal modules in SNNs

**Figure 8:**
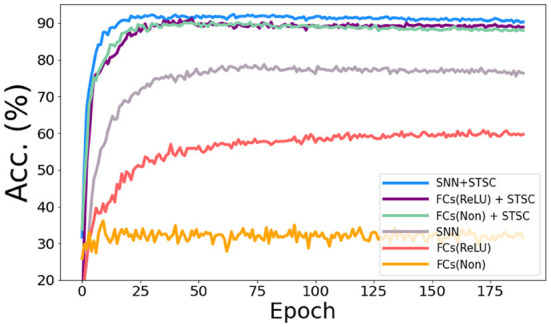
Ablation study of temporal modules in SNNs. The accuracy comparison of different models *via* training epochs on SHD. “FCs(Non)” denotes the FC structure without LIFs and activation functions, “FCs(ReLU)” denotes the FC structure with ReLU functions behind the first two FC layers, and “SNN” denotes the FC structure with LIFs behind all three FC layers. Then, STSC modules are added just behind input (P1) in three models as a comparison.

In the vanilla SNNs, only neurons perform temporal operations; hence, its temporal feature extraction is predicated solely on the temporal dependence inside each neuron. In order to assess the influence of temporal modules, we conduct the ablation study with LIF neurons and STSC modules on SHD datasets (see Figure 8), based on the same FC structure (see Table 3). Experiments comparing “FC(ReLU)” and “SNN” demonstrate that utilizing LIF neurons to replace the activation function in the FC structure can definitely increase the performance of the SHD classification task, proving the LIF's capacity to handle temporal information and capture temporal features. Moreover, the “FCs(Relu) + STSC” and “FCs(non) + STSC” structures generated by adding the STSC module obtain greater performance than the vanilla “SNN” model, demonstrating that our STSC module has superior temporal feature extraction capacity than LIF; hence, the utilization of time relationships within synaptic connections is valid and meaningful. Furthermore, integrating the STSC module and LIF concurrently inside the “SNN + STSC” model achieves the highest performance, proving that time-dependent interactions in both synapses and neurons could coexist and be coordinated to perform better temporal information processing.

## 4. Discussion

The incorporation of temporal operations inevitably increases the model's complexities and the analysis of trade-off value. Here, we explore the time and space complexity induced by the TRF and FLI modules in convolutional layers for 3D cases. Assuming the STSC modules are inserted prior to a spatial 2D convolution, the input and output tensor dimensions are *R*^*T*×*C*×*H*×*W*^ and $R^{T\times C_{out}\times H\times W}$, and the size of the convolution kernel is *O*(*K*_*c*_×*K*_*c*_). Temporal convolution (Equation 6) needs just *O*(*K*_*F*_) time complexity per element for a TRF module with a *K*_*F*_ receptive field, and the total time complexity is *O*(*T*×*C*×*H*×*W*×*K*_*F*_). For the FLI module with a *K*_*G*_ receptive field, each time slot requires a computational complexity of $O\left(C\times \frac{C}{r}\times K_{G}+C\times \frac{C}{r}\right)=O\left(C\times \frac{C}{r}\times \left(K_{G}+1\right)\right)$, and overall time complexity is $O\left(T\times C\times \frac{C}{r}\times \left(K_{G}+1\right)\right)$. In contrast to the *O*(*T*×*C*×*C*_*out*_×*H*×*W*) time complexity required for spatial 2D convolution operations, the *O*(*H*×*W*×*K*_*c*_×*K*_*c*_) and $O\left(T\times C\times \frac{C}{r}\times \left(K_{G}+1\right)\right)$ time complexity of TRF and FLI are acceptable. In addition, spatial 2D convolution needs *O*(*C*×*C*_*out*_×*K*_*c*_×*K*_*c*_) parameters, whereas TRF requires merely *O*(*C*×*K*_*F*_) parameters and FLI requires $O\left(C\times \frac{C}{r}\times \left(K_{G}+1\right)\right)$ parameters. In general, the space complexity of TRF is substantially less than that of FLI, and its additional parameters are negligible when compared to 2D convolution; the time complexity of TRF and FLI is relatively efficient in comparison to 2D convolution.

Notably, both the TRF and FLI modules are based on the sliding of time windows, and the computations for distinct time frame inputs are identical; thus, there is potential parallelism in the time dimension, and hardware implementation and optimization are possible. In the meanwhile, the computation of STSC-SNN depends on floating-point multiplication, which may reduce the energy efficiency of hardware based on the binary nature of spike transmission. Nevertheless, there is a good reason to believe that binary signals are not a strict constraint for the development of neuromorphic computing, as the carrier (electrical signal or neurotransmitter) used to transmit the spike signal in the biological synapse is not a binary information representing just presence or absence of spike activities (Rothman, 2013); in fact, a substantial amount of research has moderately loosened the binary constraint (Shrestha and Orchard, 2018; Fang et al., 2020a; Wu et al., 2021; Yao et al., 2021; Yu et al., 2022; Zhu et al., 2022). We believe that with the development of neuromorphic chips, spiking neural networks based on analog circuits and in-memory computing will be capable of surpassing the binary constraints and reconcile the biological plausibility and computational complexity of synaptic operations (Roy et al., 2019; Fang et al., 2021a; Tao et al., 2021).

## 5. Conclusion

In conclusion, this work proposes to endow synaptic structures with spatio-temporal receptive fields and additional temporal dependencies in an effort to enhance the temporal information processing capabilities of SNNs. We propose the STSC module from the standpoints of both computational models and biological realities, which consists of TRF and FLI, implemented with temporal convolution and attention mechanisms. We verified the method's reliability on neuromorphic datasets of SHD, N-MNIST, CIFAR10-DVS, and DVS-Gesture. Notably, the STSC supports SNNs in reaching the SOTA result (92.36%) on the SHD dataset, which is comparable to ANNs' methods (89 and 92.4%), validating the potential of SNNs in the spatio-temporal data processing.

## Data availability statement

The original contributions presented in the study are included in the article/supplementary material. The code is available at the following link: https://github.com/Tab-ct/STSC-SNN. Further inquiries can be directed to the corresponding author.

## Author contributions

CY proposed the idea. CY and ZG designed and did the experiments. CY, ZG, and AW wrote the manuscript. DL, GW, AW, and EL revised the manuscript. AW directed the projects and provided overall guidance. All authors contributed to the article and approved the submitted version.
